# Fluoroquinolone-Resistant *Mycoplasma genitalium,* Southwestern France

**DOI:** 10.3201/eid2209.160446

**Published:** 2016-09

**Authors:** Chloé Le Roy, Nadège Hénin, Sabine Pereyre, Cécile Bébéar

**Affiliations:** University of Bordeaux, Bordeaux, France (C. Le Roy, N. Hénin, S. Pereyre, C. Bébéar);; Institut National de la Recherche Agronomique, Villenave d'Ornon, France (C. Le Roy, N. Hénin, S. Pereyre, C. Bébéar);; Bordeaux University Hospital, Bordeaux (S. Pereyre, C. Bébéar)

**Keywords:** *Mycoplasma genitalium*, fluoroquinolones, macrolides, antimicrobial resistance, bacteria, France

**To the Editor:** *Mycoplasma genitalium* is a sexually transmitted bacterium involved in nongonococcal urethritis in men and associated with cervicitis and pelvic inflammatory disease in women. Azithromycin regimens have been commonly used as a first-line treatment of these conditions, but a recent increase in *M. genitalium* with azithromycin resistance has been described worldwide; in 2012, resistance in the organism was detected in France at a prevalence of 14% (*1*). In case of azithromycin failure, moxifloxacin is a second-line treatment; however, moxifloxacin treatment failures have also been reported and are associated with mutations in ParC or GyrA (*2*).

Prevalence of *M. genitalium* infection was ≈4% in 2013–2014 at Bordeaux University Hospital (Bordeaux, France). To evaluate the prevalence of fluoroquinolone resistance in *M. genitalium* in southwestern France, we examined the quinolone resistance–determining regions (QRDRs) of the *gyr*A and *par*C genes in *M. genitalium*–positive specimens obtained during 2013–2014. We retrospectively collected (from the Department of Bacteriology, Bordeaux University Hospital) 369 *M. genitalium*–positive urogenital specimens and DNA extracts from 344 patients. The *gyr*A and *par*C QRDRs were amplified and sequenced as described (*3**,**4*). We also assayed macrolide resistance–associated mutations using real-time PCR and melting curve analysis (*1*). To determine resistant genotypes A2058G or A2059G, we sequenced PCR products. Nucleotide positions in the 23S rRNA and amino acid positions in GyrA and ParC were identified according to *Escherichia coli* numbering.

From the 344 *M. genitalium*–positive patients, 200 specimens underwent complete analysis for the *gyr*A and *par*C genes, specimens from 221 patients were investigated for macrolide resistance, and specimens from 168 patients were examined for 23S rRNA, *gyr*A, and *par*C genes. Unsuccessful amplifications could be attributed to low bacterial loads of *M. genitalium* or to the degradation of frozen DNA during storage. Strains from 12/200 patients (6%; 95% CI 3.47%–10.19%) had QRDR mutations, with rates of 6.4% (6/93) for 2013 and 5.6% (6/107) for 2014. This prevalence is in accordance with the 4.5% moxifloxacin resistance described in the United Kingdom in 2011 (*3*) but lower than prevalences found in small numbers of strains in Japan and Australia during 2006–2014, which ranged from 10% to 47% (*4**–**8*).

Strains from 11 patients (patient nos. 6, 8, 12, 20, 23, 28–31, 46, 47) harbored alterations in the ParC QRDR (Table) at positions 80 (Ser→Asn or Ile) or 84 (Asp-84→Tyr or Asn). These mutations have been previously described for *M. genitalium* (*4**,**6**–**8*). In addition, 1 new amino acid alteration, Asn-96→Ser (strain from patient 20), was found in ParC. We detected a GyrA modification with the Ala-93→Thr transition in a strain from 1 patient (patient 3). These 2 amino acid changes were not previously reported; however, mutations at the next positions (97 in ParC and 95 in GyrA) have been described for *M. genitalium* (*4**,**7*), and these positions are within the QRDRs, suggesting their involvement in fluoroquinolone resistance. As previously described, *M. genitalium* ParC alterations predominate over GyrA alterations.

**Table:** Fluoroquinolone resistance–associated amino acid changes in GyrA and ParC and macrolide resistance–associated mutations in the 23S rRNA gene in *Mycoplasma genitalium,* France, 2013–2014*

Patient no.	Date of collection	Patient sex	Specimen type	Mutation in the 23S rRNA gene	Amino acid changes in	
					GyrA	ParC
1	2013 Jan 9	F	Vaginal swab	A2058G/A2059G	NA	NA
2	2013 Feb 1	F	Vaginal swab	A2058G	–	–
3	2013 Feb 1	M	Urethral swab	A2058G/A2059G	Ala-93→Thr	–
4	2013 Feb 18	M	Urethral swab	A2058G/A2059G	–	–
5	2013 Feb 20	M	Urine	A2058G/A2059G	–	–
6	2013 Mar 11	M	Urine	A2058G/A2059G	–	Asp-84→Tyr
7	2013 Mar 28	F	Vaginal swab	A2058G/A2059G	NA	–
8	2013 Apr 3	M	Urine	Wild-type	–	Asp-84→Tyr
9	2013 Apr 8	F	Vaginal swab	A2058G/A2059G	–	–
10	2013 Apr 11	F	Vaginal swab	A2058G/A2059G	NA	NA
11	2013 Apr 16	F	Vaginal swab	A2058G	–	NA
12	2013 Apr 19	F	Vaginal swab	Wild-type	–	Ser-80→Asn
13	2013 May 21	M	Urethral swab	A2059G	–	–
14	2013 Jul 4	M	Urine	A2059G	–	–
15	2013 Jul 4	M	Urethral swab	A2059G	–	–
	2013 Jul 19	M	Urine	A2059G	–	–
16	2013 Jul 19	M	Urine	A2058G	–	–
17	2013 Aug 9	F	Vaginal swab	A2059G	NA	–
18	2013 Sep 30	F	Vaginal swab	A2058G/A2059G	–	–
19	2013 Sep 30	F	Vaginal swab	A2058G/A2059G	–	–
20	2013 Oct 29	F	Vaginal swab	Wild-type	–	Asn-96→Ser
21	2013 Nov 22	F	Vaginal swab	A2058G/A2059G	NA	NA
22	2013 Nov 29	F	Vaginal swab	A2062T	–	–
23	2013 Dec 1	F	Vaginal swab	Wild-type	–	Asp-84→Tyr
24	2014 Jan 21	F	Vaginal swab	A2059G	–	–
25	2014 Jan 29	F	Vaginal swab	A2059G	–	–
26	2014 Jan 30	F	Vaginal swab	A2058G/A2059G	NA	–
27	2014 Feb 13	F	Vaginal swab	A2059G	–	–
28	2014 Feb 18	M	Urine	Wild-type	–	Ser-80→Ile
29	2014 Feb 24	F	Vaginal swab	Wild-type	–	Asp-84→Asn
30	2014 Mar 5	F	Vaginal swab	Wild-type	–	Asp-84→Asn
31	2014 Mar 14	M	Urine	NA	–	Ser-80→Asn
32	2014 Apr 3	F	Endocervical swab	A2058G	–	–
33	2014 Apr 7	M	Urethral swab	A2059G	–	–
34	2014 Jun 24	F	Vaginal swab	A2059C	–	–
35	2014 Jul 9	F	Endocervical swab	A2059G	NA	–
36	2014 Jul 25	F	Urine	A2058G/A2059G	NA	NA
37	2014 Jul 25	F	Vaginal swab	A2058G	–	–
38	2014 Aug 19	F	Endocervical swab	A2062T	–	NA
39	2014 Aug 28	F	Vaginal swab	A2058G/A2059G	–	–
40	2014 Sep 24	F	Vaginal swab	A2059G	–	–
41	2014 Oct 7	F	Vaginal swab	A2059G	–	–
42	2014 Oct 15	F	Vaginal swab	A2058G/A2059G	–	–
43	2014 Oct 31	M	Urine	A2058G	–	–
44	2014 Nov 5	F	Vaginal swab	A2058G/A2059G	NA	–
45	2014 Nov 28	F	Vaginal swab	A2058G	–	–
46	2014 Dec 3	F	Vaginal swab	Wild-type	–	Asp-84→Asn
47	2014 Dec 3	M	Urethral swab	Wild-type	–	Asp-84→Asn
48	2014 Dec 4	M	Urine	A2059G	–	–
*A2058/A2059G indicates a macrolide–resistant (A2058G or A2059G) genotype. Positions in the 23S rRNA and in GyrA and ParC are identified according to *Escherichia coli* numbering. NA, not available; –, no amino acid change.						

None of the 12 patients with strain *par*C or *gyr*A mutations had a history of fluoroquinolone treatment. Six patients received no treatment; 4 patients received azithromycin (1 g); 2 patients received extended azithromycin (1.5 g), 1 patient after azithromycin (1 g) failure, and 1 after receiving doxycycline for 7 days. Therapeutic outcomes were not available except for 1 patient, who experienced clinical failure after 2 azithromycin treatments.

Regarding macrolide resistance, 38 of 221 patients (17.20%; 95% CI 12.79%–22.72%) had *M. genitalium* with macrolide resistance–associated 23S rRNA mutations; prevalence was 17% (19/112) for 2013 and 17.4% (19/109) for 2014. This prevalence is increasing compared to that described in France in 2012 (14%). We found 35 A→G substitutions at position 2058 or 2059, two A2062T mutations and one A2059C mutation (Table) (*1*,*9*). Notably, in patients 15 and 33, who were infected with strains with macrolide resistance–associated mutations, *M. genitalium* infection was unsuccessfully treated with azithromycin, with treatment failures after azithromycin (1 g) and extended azithromycin (1.5 g for 5 d), but moxifloxacin treatment was effective. Patient 15 had been treated 1 year earlier with azithromycin (1 g) for nongonococcal urethritis.

Among the 168 patients whose isolates were examined for the 23S rRNA, *gyr*A, and *par*C genes, strains from 2 patients (patients 3 and 6) had both macrolide- and fluoroquinolone-associated mutations (1.2%; 95% CI 0.33%–4.24%). Both patients received azithromycin (1 g), and patient 6 received additional azithromycin (1.5 g) after failure of azithromycin (1 g). Patient 6 experienced azithromycin failure again after the extended regimen. *M. genitalium* multidrug resistance is described in France at a prevalence of 1.2%, lower than prevalence described in Australia (7.5%) (*7*) and Japan (30.8%) (*10*).

In conclusion, *M. genitalium* fluoroquinolone resistance is emerging in France, with a prevalence of 6% in 2013–2014. Further, macrolide resistance also increased during this period, to a rate of 17.2%. Patients infected with *M. genitalium* strains containing both macrolide and fluoroquinolone resistance mutations associated with therapeutic failure raise concerns about untreatable *M. genitalium* infections.
